# Targeting Exportin-1 in Cancer: From Nuclear Export to Oncogenic Transcriptional Hubs

**DOI:** 10.31662/jmaj.2026-0127

**Published:** 2026-07-10

**Authors:** Seigi Oshima, Kensuke Kanaoka, Wataru Saika, Daichi Inoue

**Affiliations:** 1 Department of Cancer Pathology, Graduate School of Medicine and Frontier Biosciences, The University of Osaka, Osaka, Japan; 2 Department of Hematology, Graduate School of Medicine, Kyoto University, Kyoto, Japan; 3 Department of Respiratory Medicine and Clinical Immunology, Graduate School of Medicine, The University of Osaka, Osaka, Japan; 4 Department of Hematology, Shiga University of Medical Science, Otsu, Japan; 5 Institute for Open and Transdisciplinary Research Initiatives, The University of Osaka, Osaka, Japan

**Keywords:** exportin-1 (XPO1/CRM1), XPO1 inhibitors, nuclear export signals (NES), transcriptional hubs, nuclear condensates

## Abstract

Maintaining cellular homeostasis requires precise coordination of molecular trafficking between the nucleus and the cytoplasm. Central to this process is the nucleocytoplasmic transport system, which governs the spatial distribution of proteins and RNA. Exportin-1 (XPO1), also known as chromosome region maintenance 1 (CRM1), is the principal nuclear export receptor responsible for the transport of hundreds of cargo proteins bearing leucine-rich nuclear export signals. In a wide range of malignancies, XPO1 is frequently dysregulated through overexpression or recurrent somatic mutations. This dysregulation results in inappropriate cytoplasmic sequestration of tumor suppressor proteins and other regulatory factors, thereby facilitating oncogenic signaling and malignant transformation. Cancer cells consequently become highly dependent on XPO1-mediated nuclear export, positioning XPO1 as a rational therapeutic target. Consistent with this concept, selective inhibitors of nuclear export, such as selinexor, have demonstrated clinical efficacy, received regulatory approval for the treatment of multiple myeloma and diffuse large B-cell lymphoma, and are currently being evaluated in clinical trials for solid tumors. Beyond the canonical transport function, accumulating evidence indicates that XPO1 performs noncanonical roles as a chromatin-associated scaffold that supports the assembly of oncogenic transcriptional hubs and phase-separated biomolecular condensates. These structures integrate nuclear transport machinery with transcriptional regulatory complexes to drive aberrant gene expression programs, particularly in leukemias harboring *NPM1* mutations, *NUP98* fusions, or *UBTF* tandem duplications. Disruption of these hubs through XPO1 inhibition represents a therapeutic strategy that extends beyond simple nuclear retention of tumor suppressors, potentially reprogramming malignant transcriptional states. This review outlines the physiological roles of XPO1 as a nuclear exporter and chromatin regulator, examines the pathological consequences of its dysregulation in cancer, summarizes current therapeutic strategies and their clinical outcomes, and discusses emerging evidence for noncanonical functions of XPO1 in nuclear organization and transcriptional regulation.

## Introduction

Cells maintain homeostasis through systems that govern the spatial compartmentalization of biochemical reactions. Central to these processes is the flow of genetic information encoded in DNA, transcribed into RNA, and subsequently translated into protein, a framework commonly referred to as the central dogma. The physical separation of transcription and translation in eukaryotic cells necessitates precise regulation of molecular trafficking between the nucleus and the cytoplasm, rendering nucleocytoplasmic transport an essential determinant of cellular homeostasis.

In eukaryotic cells, mRNA synthesized in the nucleus is exported to the cytoplasm, where it is translated into protein on ribosomes. Conversely, proteins that function in the nucleus, including transcription factors and components of RNA processing machinery, are actively imported into the nucleus through nuclear import receptors known as importins. This bidirectional trafficking is tightly regulated to ensure that each protein resides in the appropriate cellular compartment at the appropriate time. Proteins that have completed their nuclear functions or that have become dysfunctional are actively exported from the nucleus and subsequently degraded or recycled, a process essential for maintaining nuclear integrity and homeostasis. Disruption of this finely tuned transport system can therefore have profound consequences for cellular function.

Exportin-1 (XPO1), also known as chromosome region maintenance 1 (CRM1), is a major nuclear export receptor that mediates the transport of cargo proteins from the nucleus to the cytoplasm. Accumulating evidence indicates that aberrant XPO1 activity contributes to the pathogenesis of various malignancies ^(1), (2), (3), (4), (5), (6), (7), (8)^. Dysregulated nuclear export results in inappropriate cytoplasmic sequestration of tumor suppressors and key regulatory proteins, thereby promoting oncogenic signaling and malignant transformation. These observations have established XPO1 as a rational therapeutic target in oncology. To date, several selective XPO1 inhibitors have advanced to clinical trials, and one received approval from the US Food and Drug Administration (FDA) for the treatment of multiple myeloma (MM) ^(9)^ and diffuse large B-cell lymphoma (DLBCL) ^(10)^.

Beyond its canonical role in nuclear export, recent studies—particularly in the context of hematologic malignancies—have revealed previously unrecognized functions of XPO1 in nuclear organization and gene regulation. XPO1 has been implicated in the formation of transcriptional hubs, liquid-liquid phase separation, and higher-order regulation of gene expression, indicating that its role extends beyond nucleocytoplasmic transport ^(11), (12)^. These emerging concepts have broadened our understanding of the nuclear export machinery as an active participant in transcriptional regulation and oncogenic gene expression programs.

In this review, we first outline the physiological role of XPO1 as a nuclear export receptor and examine how its dysregulation contributes to cancer pathogenesis. We then summarize current therapeutic strategies targeting XPO1 and their clinical implications. Finally, we highlight recent findings revealing noncanonical functions of XPO1 in transcriptional regulation and phase separation, with a particular focus on their relevance to hematologic malignancies and future therapeutic directions.

## Physiological Role of XPO1 as a Nuclear Export Receptor

XPO1 is a ring-shaped protein that mediates the nuclear export of hundreds of cargo proteins. Identified nearly three decades ago ^(13), (14), (15), (16)^, the protein has been studied primarily for its essential role as a nuclear export receptor. XPO1 comprises HEAT repeat motifs arranged in a toroidal (ring-shaped) structure that confers marked conformational flexibility, enabling interactions with a broad range of binding partners. Under physiological conditions, XPO1 recognizes cargo proteins bearing hydrophobic, leucine-rich amino acid sequences termed nuclear export signals (NES). Upon cargo recognition, XPO1 assembles a trimeric export complex together with the cargo protein and Ran-GTP in the nucleus. Binding of Ran-GTP induces a conformational change of XPO1 into an active state, thereby stabilizing cargo association and enabling directional transport through the nuclear pore complex (NPC) ^(17), (18)^. XPO1 then engages directly with phenylalanine-glycine (FG) repeat–containing nucleoporins, including NUP98 and NUP214, to facilitate translocation across the nuclear envelope ^(19), (20)^. Once the trimeric export complex reaches the cytoplasm, Ran-GTP is hydrolyzed to Ran-GDP, leading to dissociation of the trimeric complex and the release of the cargo. XPO1 is subsequently recycled to the nucleus to reenter this coordinated, multistep export cycle (Figure 1).

**Figure 1.**
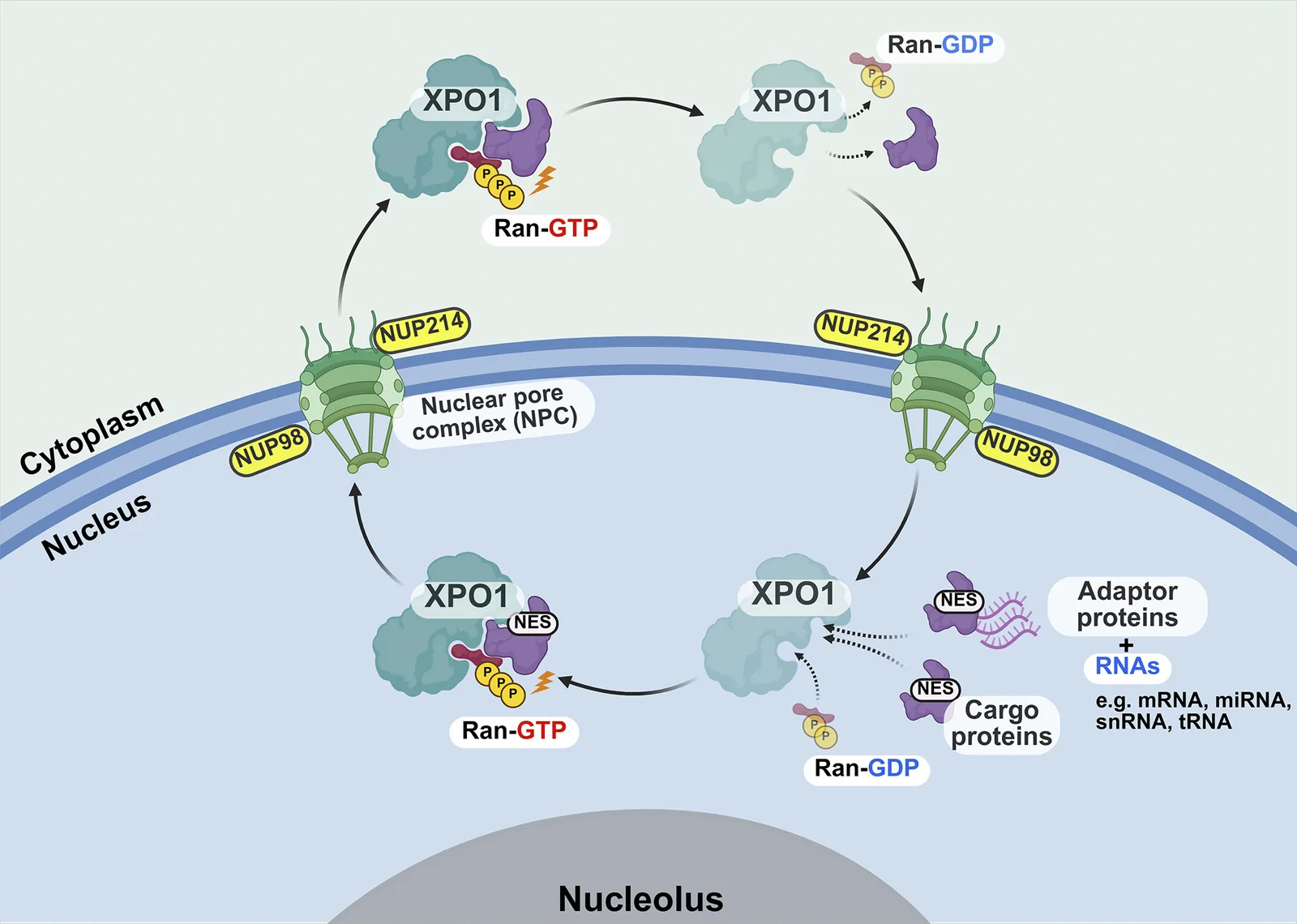
Mechanism of XPO1-mediated nuclear export. XPO1 (CRM1) exports proteins bearing leucine-rich NES from the nucleus to the cytoplasm. In the nucleus, XPO1 binds cargo proteins together with Ran-GTP to form a trimeric export complex that translocates through the NPC via interactions with FG-repeat nucleoporins such as NUP98 and NUP214. In the cytoplasm, Ran-GTP hydrolysis to Ran-GDP triggers complex dissociation and cargo release. XPO1 is then recycled to the nucleus for another round of export. In addition to protein cargo, XPO1 also mediates the export of specific RNAs via adaptor proteins. (Created with BioRender.com.) CRM1: chromosome region maintenance 1; FG: phenylalanine-glycine; miRNA: microRNA; NES: nuclear export signals; NPC: nuclear pore complex; snRNA: small nuclear RNA; tRNA: transfer RNA; XPO1: exportin-1.

Large-scale proteomic and computational studies have uncovered the breadth of the XPO1 cargo repertoire. More than 1000 proteins have been identified as XPO1 substrates, encompassing transcription factors, cell cycle regulators, and signaling molecules such as p53 and p27 ^(21)^. Dedicated databases and predictive tools, such as ValidNESs and LocNES, further underscore the prevalence of functional NES motifs across the proteome ^(22), (23)^. Beyond protein export, XPO1 contributes to RNA trafficking through adaptor proteins and has been implicated in the nuclear export and processing of specific RNA species, including subsets of mRNAs, microRNAs, small nuclear RNAs, and transfer RNAs ^(24), (25)^.

## XPO1 Dysregulation in Cancer

Accumulating evidence has implicated XPO1 in cancer biology. In hematologic malignancies, a recurrent point mutation in *XPO1* (p.E571K) has been identified in primary mediastinal B-cell lymphoma ^(26)^, and classical Hodgkin lymphoma, in which approximately 25% of patients harbor this mutation ^(27)^. Although the precise functional mechanisms by which this mutation contributes to oncogenesis remain incompletely understood, several studies have suggested that the p.E571K substitution alters the binding affinity of XPO1 for specific protein cargos ^(28), (29)^. In one study, the XPO1 p.E571K mutation altered NES recognition and nuclear/cytoplasmic compartmentalization, promoted cellular proliferation, and induced B-cell lymphomagenesis in vivo ^(29)^. XPO1 dysregulation is not restricted to hematologic cancers. Pan-cancer analyses and tumor-specific studies have documented aberrant XPO1 overexpression across a broad spectrum of solid tumors, including pancreatic, ovarian, lung, gastric, prostate, and colorectal cancers ^(3), (4), (5), (6), (7), (8)^. Elevated XPO1 expression is frequently associated with advanced tumor stage, aggressive clinicopathological features, and poor overall survival (OS), further supporting a functional role for XPO1 dysregulation in tumor progression ^(30), (31), (32)^.

A prevailing model proposes that XPO1 overexpression or functional hyperactivation enhances the nuclear export of critical tumor suppressor proteins, thereby promoting oncogenesis. XPO1 substrates include several well-characterized tumor suppressors, such as p53, BRCA1, BRCA2, RB1, APC, and p27, whose nuclear retention is essential for their tumor-suppressive functions. Aberrant XPO1-mediated export of these factors to the cytoplasm results in their functional inactivation, providing a mechanistic basis for XPO1-driven tumor progression (Figure 2).

**Figure 2.**
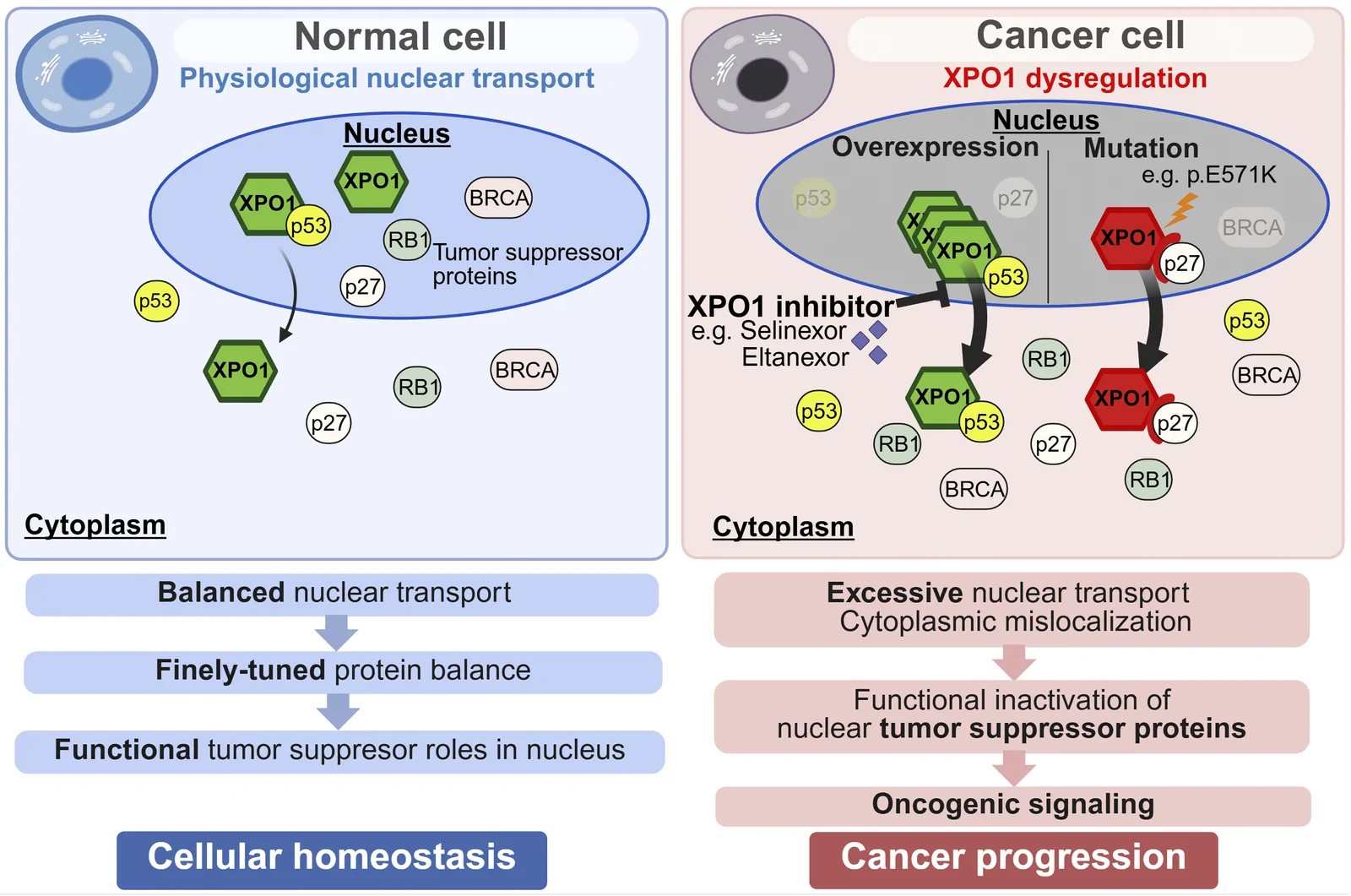
XPO1 dysregulation in cancer. In normal cells, XPO1 maintains balanced nuclear export, ensuring proper localization of tumor suppressor proteins such as p53, p27, RB1, and BRCA. In cancer cells, XPO1 overexpression or mutation (e.g., p.E571K) enhances nuclear export, leading to cytoplasmic mislocalization and functional inactivation of tumor suppressor proteins, thereby promoting oncogenic signaling and cancer progression. Pharmacological inhibition of XPO1, such as with selinexor or eltanexor, restores nuclear retention of these proteins and counteracts tumor-promoting signaling. (Created with BioRender.com.) XPO1: exportin-1.

## Targeting the Canonical Nuclear Export Function of XPO1

Building on the established role of XPO1 in nuclear export and its dysregulation in cancer, pharmacological inhibition of XPO1 has emerged as a rational therapeutic strategy. The first compound identified as a nuclear export inhibitor was leptomycin B (LMB), a natural product originally isolated as an antifungal agent ^(33)^. LMB covalently binds to Cys528 located within the NES recognition groove of XPO1, thereby preventing interactions between XPO1 and its cargo proteins ^(34)^. Structural and biochemical analyses revealed that this irreversible conjugation results in sustained inhibition of nuclear export. In preclinical studies, LMB demonstrated potent antitumor activity in xenograft and transplantation mouse models, providing early proof-of-concept that XPO1 is a therapeutically tractable target. Despite these promising preclinical findings, LMB failed in clinical development: a phase I trial was discontinued owing to severe dose-limiting toxicities, including anorexia and vomiting, with minimal clinical benefit. Subsequent efforts led to the development of selective inhibitors of nuclear export (SINEs), a new class of small molecules specifically designed to address the limitations of LMB. Like LMB, SINE compounds target Cys528 within the cargo-binding groove of XPO1 ^(35)^; however, they form a slowly reversible covalent bond, in contrast to the irreversible adduct formed by LMB. This reversible binding substantially improves tolerability and permits repeated dosing, facilitating successful clinical translation. Preclinical studies demonstrated that SINE compounds selectively induce apoptosis in malignant cells while largely sparing normal hematopoietic cells ^(36)^. At the molecular level, XPO1 inhibition results in nuclear retention of multiple tumor suppressor proteins, including p53 and p27, and disrupts oncogenic signaling pathways and transcriptional programs ^(37), (38)^. Among SINE compounds, selinexor (KPT-330) was the first to enter clinical trials and demonstrated measurable clinical benefit in patients with relapsed or refractory hematologic malignancies, including MM and DLBCL, leading to approval by the FDA ^(9), (10)^. Second-generation SINEs, including eltanexor (KPT-8602), were developed to improve tolerability through reduced central nervous system penetration and are currently under evaluation across a range of hematologic and solid tumors. Notably, the reduced dosing constraints of eltanexor enable more sustained XPO1 inhibition, which has been associated with prolonged suppression of *HOX* gene expression and superior antileukemic activity compared with selinexor in preclinical models ^(39)^.

### Clinical trials in hematologic malignancies

Clinical validation of XPO1 inhibition has been most extensively established in hematologic malignancies, culminating in the first regulatory approvals of selinexor. In relapsed or refractory MM, the pivotal phase IIb STORM trial (Part 2) evaluated selinexor (80 mg) in combination with low-dose dexamethasone in heavily pretreated patients who had been exposed to the three major therapeutic classes—proteasome inhibitors, immunomodulatory drugs, and anti-CD38 monoclonal antibodies—and were refractory to these treatments (penta-exposed, triple-class refractory patients) ^(9)^. The trial reported an overall response rate (ORR) of 26%, with a median duration of response of 4.4 months and OS of 8.6 months, establishing selinexor as a therapeutic option for this otherwise treatment-exhausted population. In the subsequent randomized phase III BOSTON trial, the addition of selinexor to once-weekly bortezomib and dexamethasone (XVd) significantly prolonged progression-free survival (PFS) compared with bortezomib and dexamethasone (Vd) alone (median PFS: 13.9 vs. 9.5 months; hazard ratio [HR] 0.70; p = 0.0075) ^(40)^. The ORR was 76% in the XVd group compared with 62% in the Vd group (p = 0.0012). The safety profile was manageable, with the most common grade 3 or higher adverse events being thrombocytopenia (39%), anemia (16%), and fatigue (13%). Notably, the XVd regimen employed a reduced bortezomib dosing schedule and was associated with a lower incidence of peripheral neuropathy, suggesting that optimized combination strategies may improve the tolerability of selinexor-based therapy ^(40)^.

In relapsed or refractory DLBCL, the phase IIb SADAL trial investigated selinexor monotherapy in patients who had received at least two prior lines of systemic therapy ^(10)^. The median age was 67 years (range 35–87), and 57 patients (45%) were aged ≥70 years. The trial reported an ORR of 28%, including a 12% complete response rate, with responses observed across histological subtypes, including germinal center B-cell (GCB) and non-GCB subtypes. The median duration of response was 9.3 months and was longer among patients who achieved complete responses (23.0 months). The most common adverse events were thrombocytopenia, neutropenia, anemia, and fatigue, consistent with those reported in previous clinical trials. These results were noteworthy given the oral administration and the manageable safety profile in a heavily pretreated elderly population.

Beyond MM and DLBCL, clinical investigation has expanded to other lymphoid and myeloid neoplasms. In myelofibrosis, the ongoing phase III SENTRY trial (NCT04562389) is evaluating selinexor in combination with ruxolitinib, based on phase I/II data demonstrating meaningful reductions in spleen volume and symptom burden ^(41)^. In myelodysplastic syndromes, a phase I/II clinical trial (NCT05918055) is currently evaluating the combination of eltanexor with the oral hypomethylating agent decitabine-cedazuridine in patients with higher-risk disease, with the aim of determining the recommended phase II dose and assessing the safety and efficacy of this combination in adult patients. In acute myeloid leukemia (AML), several early-phase trials are exploring the therapeutic potential of selinexor and eltanexor either as monotherapy or in combination with other targeted agents. Representative clinical trials are listed in Table 1 ^(9), (10), (40), (41), (42), (43), (44), (45)^. Collectively, these landmark trials demonstrate that XPO1 inhibition, whether as monotherapy or in rational combinations, can overcome treatment resistance across a broad spectrum of hematologic malignancies.

**Table 1.** Phase II/III Clinical Trials of XPO1 Inhibitors in Hematologic Malignancies.

Study/ID	Phase	Disease entity	Regimen	Key outcomes/findings	Reference
STORM (Part 2)	IIb	RRMM (penta-exposed, triple class RRMM)	Selinexor + dexamethasone	ORR 26%, mDOR 4.4 mo; FDA approval for RRMM	^(9)^
BOSTON	III	RRMM (≥1 prior line)	Selinexor + bortezomib + dexamethasone	mPFS 13.9 (XVd) vs. 9.5 mo (Vd) (HR 0.70; p = 0.0075), ORR 76% (XVd) vs. 62% (Vd) (p = 0.0012); weekly dosing improved tolerability	^(40)^
MARCH	II	RRMM (refractory to PI and IMiDs)	Selinexor + dexamethasone	ORR 29% (25% in triple-class RRMM), mDOR 4.7 mo, mPFS 3.7 mo, mOS 13.2 mo	^(42)^
SADAL	IIb	RR DLBCL (≥2 prior lines)	Selinexor monotherapy	ORR 28%, CR 12%, mDOR 9.3 mo; safety profile manageable in a heavily treated elderly population	^(10)^
SENTRY XPORT-MF-034	I/III	Myelofibrosis (JAKi-naïve)	Selinexor + ruxolitinib	Ongoing: double-blind, placebo-controlled; evaluate SVR35 and TSS50; Phase 1 data showed 79% and 58%, respectively	^(41)^
NCT02228525	II	High-risk MDS/AML refractory to HMA	Selinexor monotherapy	ORR 26%, SD 39%, mDOR 6.3 mo, mOS 8.7 mo; selinexor showing response to HMA-refractory MDS/AML	NA
NCT05918055	I/II	High-risk MDS	Eltanexor + decitabine-cedazuridine	Ongoing: determine ORR of eltanexor + HMA combination therapy	NA
NA	Ib/II	Newly diagnosed MDS-EB-1/2	Selinexor + azacitidine	ORR 94%, CR 39% (*TP53*-mutated, ORR 100%, CR 47%), mPFS 24.2 mo, 2-y OS 84%, 2-y PFS 50%	^(43)^
NCT05736965	II	Newly diagnosed unfit AML	Selinexor + azacitidine + venetoclax	ORR 90%, CRc 80%, (ELN 2022 classification: adverse risk, ORR 89%, CRc 83%), MRD-negativity, 49%	^(44)^
NA	II	Newly diagnosed AML/MDS	Selinexor + azacitidine + venetoclax	CR/CRi 69%, PR 24%	^(45)^
NCT07437170	II	Pediatric NUP98-r AML (aged 1–18 y)	Selinexor + intensive chemotherapy	Ongoing	NA
SELHEM-2 NCT03071276	I/II	RR AML (aged ≤24 y)	Selinexor, fludarabine, cytarabine	ORR 54%, CR/CRi 50%	NA

AML: acute myeloid leukemia; CR: complete response; CRi: complete remission with incomplete hematologic recovery; CRc: composite complete remission; FDA: Food and Drug Administration; ELN: European LeukemiaNet; HMA: hypomethylating agent; HR: hazard ratio; IMiDs: immunomodulatory drugs; JAKi: Janus kinase inhibitor; mDOR: median duration of response; MDS: myelodysplastic syndromes; MDS-EB: myelodysplastic syndromes with excess blasts; mOS: median overall survival; mPFS: median progression-free survival; MRD: minimal residual disease; NA: not available/applicable; NUP98-r: NUP98-rearrangement; ORR: objective response rate; PI: proteasome inhibitor; PR: partial response; RR DLBCL: relapsed/refractory diffuse large B-cell lymphoma; RRMM: relapsed/refractory multiple myeloma; SD: stable disease; SVR35: spleen volume reduction ≥35%; TSS50: total symptom score ≥50%; Vd: bortezomib + dexamethasone; XPO1: exportin-1; XVd: selinexor + bortezomib + dexamethasone.

### Clinical trials in solid tumors

Clinical evidence supporting XPO1 inhibition in solid tumors has accumulated across multiple disease entities. In dedifferentiated liposarcoma, the randomized phase II/III SEAL trial demonstrated a modest but statistically significant improvement in PFS with selinexor compared with placebo (median PFS 2.8 vs. 2.1 months; HR 0.70; p = 0.011); however, OS did not differ significantly between arms, a finding attributed in part to the crossover design ^(46)^. An exploratory analysis identified CALB1 expression as a potential biomarker of resistance.

In endometrial cancer, the phase III SIENDO trial evaluated selinexor as maintenance therapy following response to platinum–taxane chemotherapy. The primary end point was not met in the intention-to-treat population (median PFS 5.7 vs. 3.8 months; HR 0.76; p = 0.13); however, a prespecified exploratory analysis revealed a substantial PFS benefit in patients with *TP53* wild-type tumors (median PFS 13.7 vs. 3.7 months; HR 0.41; p = 0.002), whereas no benefit was observed in *TP53*-mutant disease. These findings position *TP53* mutational status as a candidate predictive biomarker for selinexor maintenance therapy ^(47)^, and a confirmatory randomized phase III trial enriching for *TP53* wild-type endometrial cancer is currently ongoing ^(48)^.

Beyond these phase III data, numerous phase I and II studies of XPO1 inhibitors have been conducted across a range of solid tumor types; phase II trials, together with the aforementioned phase III trials, are summarized in Table 2 ^(46), (47), (48), (49), (50), (51), (52), (53), (54)^. Although objective response rate with XPO1 inhibitor monotherapy has generally been modest, combination strategies incorporating cytotoxic chemotherapy or immune checkpoint inhibitors are under active investigation ^(55), (56)^. Currently, there are more than 100 ongoing clinical trials evaluating the efficacy of selinexor in a wide variety of tumors (ClinicalTrials.gov). Taken together, these data support XPO1-mediated nuclear export as a clinically relevant therapeutic target across both hematologic and solid malignancies, while highlighting the need for predictive biomarkers to optimize patient selection.

**Table 2.** Phase II/III Clinical Trials of XPO1 Inhibitors in Solid Tumors.

Study/ID	Phase	Disease entity	Regimen	Key outcomes/findings	Reference
NCT02215161	II	Prostate cancer (refractory to abiraterone and/or enzalutamide)	Selinexor	Prostate-specific antigen response (≥50% decline): 14%; radiographic response (RECIST): PR 25%, SD 50%	^(49)^
NCT02402764	II	Triple-negative breast cancer (heavily pretreated; median 2 prior chemotherapy lines)	Selinexor	ORR: 0%; mPFS: 0.92 mo, mOS: 5.98 mo	^(50)^
NCT02025985	II	Gynecological cancer (heavily pretreated; median prior regimens—ovarian: 6, endometrial: 2, cervical: 3)	Selinexor	DCR (≥12 wk)—total: 30%, ovarian: 30%, endometrial: 35%, cervical: 24%; confirmed PR—ovarian: 8%, endometrial: 9%, cervical: 4%	^(51)^
KING	II	Glioblastoma (recurrence/progression after radiation and temozolomide, KPS ≥60)	Selinexor; arm B: 50 mg/m^2^ twice weekly; arm C: 60 mg flat dose twice weekly; arm D: 80 mg flat dose once weekly	6-mo PFS—arm B: 10.0%, arm C: 7.7%, arm D: 17.2%; mOS—arm B: 10.5 mo, arm C: 8.5 mo, arm D: 10.2 mo	^(52)^
NCT03095612	I/II	*KRAS*-mutant advanced NSCLC (advanced, previously treated with ≥1 prior line)	Selinexor + docetaxel	ORR: 22%; mPFS—KRAS^G12C^: 4.1 mo, KRAS^non-G12C^: 4.0 mo	^(53)^
NCT03193437/NCT03466827	II	Thymoma and thymic carcinoma (progression after ≥1 platinum-containing chemotherapy regimen)	Selinexor	ORR—thymoma: 12.5%, thymic carcinoma: 6.7%; mPFS—thymoma: 13.6 mo, thymic carcinoma: 7.8 mo; mOS—thymoma: not reached, thymic carcinoma: 15.5 mo	^(54)^
SEAL	III	DD-LPS (advanced unresectable; 2–5 prior therapies)	Selinexor (vs. placebo)	mPFS: 2.8 mo vs. 2.1 mo (HR 0.70, p = 0.011); time to next treatment: 5.8 mo vs. 3.2 mo (HR 0.50, p < 0.0001); mOS: 10.0 mo vs. 12.9 mo (HR 1.02, p = 0.54)	^(46)^
SIENDO	III	Endometrial cancer (after response to platinum–taxane chemotherapy)	Selinexor (vs. placebo)	mPFS: 5.7 mo vs. 3.8 mo (HR 0.76, p = 0.126); *TP53* wild-type subgroup PFS: 13.7 mo vs. 3.7 mo (HR 0.41, p = 0.002)	^(47)^
NCT05611931	III	Endometrial cancer (*TP53* wild-type advanced or recurrent; CR/PR after ≥12 wk of platinum-based therapy ± immunotherapy)	Selinexor	Ongoing	^(48)^

CR: complete response; DCR: disease control rate; DD-LPS: dedifferentiated liposarcoma; HR: hazard ratio; KPS: Karnofsky performance status; ORR: objective response rate; mOS: median overall survival; mPFS: median progression-free survival; NSCLC: non–small cell lung cancer; PFS: progression-free survival; PR: partial response; RECIST: Response Evaluation Criteria in Solid Tumors; SD: stable disease; XPO1: exportin-1.

## Emerging Roles of XPO1 as an Oncogenic Transcriptional Hub

Beyond its classical role as a nuclear export receptor, XPO1 has increasingly been recognized to serve pleiotropic functions. Early studies revealed that XPO1 participates in fundamental processes of cell division, including mitotic regulation, centrosome duplication, and microtubule organization. During mitosis, XPO1 forms a complex with nucleophosmin (NPM1) and is recruited to centrosomes in a Ran-GTP–dependent manner, where it contributes to centrosome integrity and microtubule assembly ^(57)^. XPO1 also contributes to proper centromere and kinetochore assembly by binding to the chromosomal passenger protein survivin through its NES motif, a process required for accurate survivin localization to centromeres. Disruption of the XPO1–survivin interaction results in defective spindle-kinetochore attachments and impaired chromosome segregation ^(58)^. Taken together, these observations indicate that XPO1 plays active roles in the spatial organization of mitotic structures, independently of its canonical nuclear export function.

More recently, XPO1 has been implicated in the organization of higher-order nuclear architecture and gene regulatory programs. Rather than functioning solely as a transport factor, XPO1 can associate with chromatin and act as a scaffold supporting the assembly of oncogenic transcriptional hubs, particularly in hematologic malignancies (Figure 3). This promotes aberrant gene expression programs through chromatin association and nuclear condensate formation.

**Figure 3.**
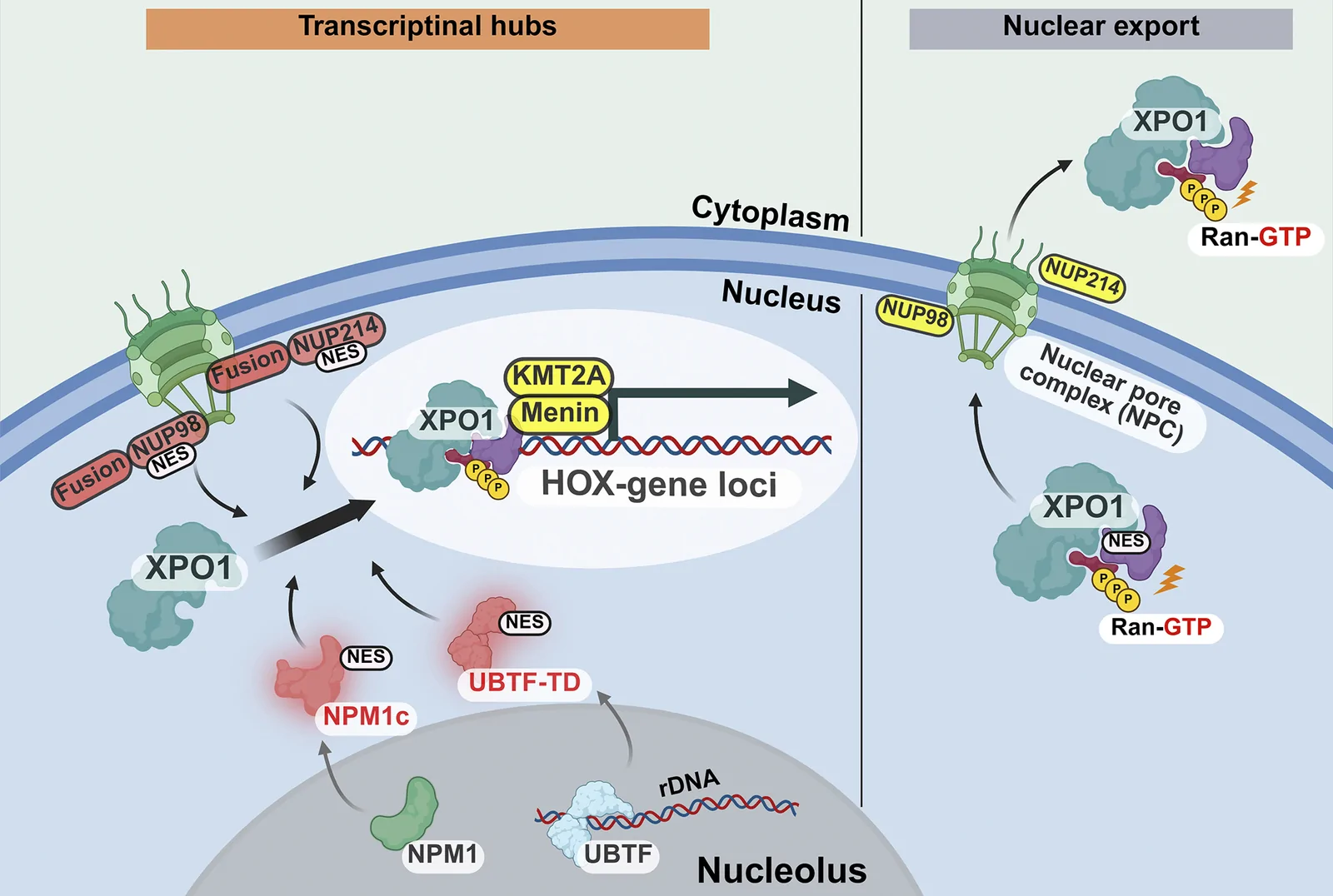
Emerging roles of XPO1 as an oncogenic transcriptional hub. Beyond its canonical role in nuclear export, XPO1 can associate with chromatin and participate in the formation of oncogenic transcriptional hubs. In leukemias harboring nucleoporin fusions (e.g., NUP98 or NUP214), *NPM1* mutations (NPM1c), or *UBTF* tandem duplications (*UBTF*-TD), newly generated or exposed NES enable aberrant interactions with XPO1. XPO1 recruits these oncogenic factors to *HOX* gene loci, where they cooperate with transcriptional regulators such as KMT2A and Menin to drive aberrant activation of *HOX* gene expression. (Created with BioRender.com.) NES: nuclear export signals; NPC: nuclear pore complex; XPO1: exportin-1.

A mechanistic study in PICALM::MLLT10 leukemia provided the first direct evidence supporting this notion. XPO1 was shown to bind directly to *HOXA* chromatin loci and recruit the PICALM::MLLT10 fusion protein, thereby driving aberrant activation of *HOXA* cluster genes ^(59)^. Notably, PICALM::MLLT10 mutants incapable of binding XPO1 failed to associate with chromatin and could not activate *HOXA* transcription, indicating that XPO1 is required for tethering the fusion protein to its target genomic loci. Subsequent studies expanded this paradigm by demonstrating that chromatin-bound XPO1 interacts with nuclear transport proteins, including FG-rich nucleoporins such as NUP98 and NUP214, which themselves are recurrently involved in leukemia-associated fusion genes. Chromatin-bound XPO1 was shown to recruit the NUP98::HOXA9 fusion protein to *HOX* cluster genes, inducing aberrant *HOX* expression and leukemic transformation ^(60)^. These findings are consistent with observations in yeast showing that XPO1 binds both transcription factors and nuclear pore proteins to dock chromatin at the NPC, thereby promoting transcriptional activation ^(61)^. Collectively, these observations position XPO1 as a chromatin-associated organizer that links oncogenic fusion proteins, nuclear transport machinery, and transcriptionally active genomic regions (Figure 3).

Extending this model, recent studies have demonstrated that XPO1 participates in the formation of biomolecular condensates through liquid-liquid phase separation. Nuclear bodies formed by nucleoporin fusion proteins promote condensation of transcriptional regulators, including KMT2A and XPO1, leading to large-scale reorganization of three-dimensional genome architecture ^(62)^. Consistent with this model, a range of leukemia-associated alterations, including *KMT2A*-rearranged leukemias and leukemias harboring NUP98 or NUP214 fusions (such as *NUP98::NSD1*, *SET::NUP214*, *DEK::NUP214*, and *SQSTM1::NUP214*), converge on phase-separated nuclear condensates that drive aberrant *HOX* gene expression and sustain oncogenic transcriptional programs ^(63), (64), (65), (66)^. In each of these contexts, XPO1 plays a central role in sustaining aberrant *HOXA* and *MEIS1* expression. These phase-separated environments may act as molecular filters that selectively concentrate transcriptional machinery while excluding inhibitory factors.

XPO1-dependent transcriptional hub formation is not restricted to nucleoporin fusion–driven leukemias. *NPM1*-mutant AML represents one of the most prevalent molecular subtypes, accounting for approximately one-third of AML cases ^(67)^. The majority of *NPM1* mutations involve 4-bp insertions in exon 12, causing a frameshift that generates a novel C-terminal NES. This acquired NES mediates aberrant binding of the mutant protein (NPM1c) to XPO1, which in turn recruits NPM1c to *HOX* cluster genes, resulting in robust *HOX* gene expression and leukemogenesis ^(68), (69), (70)^. Pharmacological inhibition of XPO1 disrupts this chromatin association and suppresses *HOX* gene expression. A mechanistically analogous role for XPO1 has recently been identified in AML driven by *UBTF* tandem duplication (UBTF-TD). In this subtype, a duplication within exon 13 of *UBTF* alters protein structure and exposes a cryptic leucine-rich NES motif, enabling aberrant interaction with XPO1. Whereas wild-type UBTF is a nucleolar protein involved in ribosomal DNA transcription, UBTF-TD is ectopically recruited to *HOX* chromatin loci via XPO1 and, together with KMT2A and Menin, drives aberrant activation of *HOX* and *MEIS* genes, contributing to leukemogenesis ^(71)^. Treatment with the XPO1 inhibitor eltanexor reduced the chromatin occupancy of UBTF-TD at *HOX* loci and suppressed leukemia progression in preclinical models ^(72)^. Taken together, *NPM1*-mutant and UBTF-TD AML illustrate a unifying pathogenic principle in which somatic mutations generate or expose NES motifs, enabling XPO1-mediated assembly of ectopic transcriptional hubs on chromatin and aberrant activation of leukemia-associated gene programs (Figure 3).

The oncogenic transcriptional hubs and phase-separated nuclear condensates assembled in these leukemias share core regulatory components, including XPO1, Menin, and KMT2A complexes, which cooperatively sustain aberrant *HOX* and *MEIS* transcriptional programs within oncogenic condensates. Leukemia cells exhibit a marked dependency on these transcriptional hubs, rendering them selectively vulnerable to their disruption. In this context, Menin inhibitors exemplify this therapeutic principle: pharmacological disruption of the Menin–KMT2A interaction downregulates *HOX* gene expression and has demonstrated clinical efficacy in *KMT2A*-rearranged and *NPM1*-mutant AML, leading to FDA approval ^(66), (73), (74)^. These clinical advances establish transcriptional hub dependency as a therapeutically actionable vulnerability in leukemia and support XPO1 inhibition as a complementary approach targeting the same oncogenic regulatory architecture.

In summary, accumulating evidence redefines XPO1 as a multifunctional regulator that integrates nuclear transport, chromatin organization, phase separation, and oncogenic transcriptional control. This broader mechanistic understanding provides a conceptual framework for the selective vulnerability of certain cancers, particularly leukemia, to XPO1 inhibition and positions transcriptional hub disruption as a rational therapeutic strategy.

## Conclusion/Future Perspectives

The understanding of XPO1 has shifted from its role as a simple nuclear export protein to a multifunctional regulator of the 3D genome and oncogenic transcription. Its canonical function in nucleocytoplasmic transport remains a primary therapeutic target, as validated by the approval of SINE compounds in hematologic malignancies and promising results in solid tumors such as endometrial cancer. However, the discovery of noncanonical roles in organizing transcriptional hubs and nuclear condensates introduces a new dimension to its oncogenic potential.

Future research must elucidate the molecular basis for the preferential recruitment of XPO1 to specific genomic regions, such as *HOX* cluster genes, and determine how diverse cargo proteins are selectively utilized by cancer cells. Furthermore, the development of biomarkers, such as *TP53* wild-type status in endometrial cancer and *CALB1* expression in liposarcoma, will be essential for the rational selection of patients. Answering these questions will facilitate the design of next-generation therapeutic strategies that target the integrated network of nuclear transport, chromatin organization, and phase separation in cancer.

## Article Information

### Acknowledgments

The authors used ChatGPT and Gemini to assist with English language editing and improve the readability of the manuscript. The authors reviewed and modified, if necessary, all content and take full responsibility for the content of the publication. Figures 1–3 were created using BioRender.com.

### Author Contributions

Seigi Oshima and Kensuke Kanaoka contributed equally to this work. Seigi Oshima and Kensuke Kanaoka conceived the study and wrote the manuscript with the support of Wataru Saika and Daichi Inoue. Seigi Oshima prepared the figures and Table 1, and Kensuke Kanaoka prepared Table 2. Wataru Saika and Daichi Inoue critically revised the manuscript and provided intellectual input. All authors reviewed and approved the final version of the manuscript.

### Conflicts of Interest

Daichi Inoue has received research funding from Sumitomo Pharma and Mitsubishi Tanabe Pharma Corporation. The other authors declare no conflicts of interest.

### Article Information

This article is based on the study that received the Medical Research Encouragement Prize of the Japan Medical Association in 2025.
